# Comparative analysis of *Microtus fortis* and murine hosts reveals a correlation between BRD4 and hepatic inflammation during *Schistosoma japonicum* infection

**DOI:** 10.1186/s13071-025-06821-z

**Published:** 2025-07-04

**Authors:** Ming Yuan, Mingrou Wu, Yunyi Hu, Siyu Zhao, Jehangir Khan, Zhanhong Yuan, Yun Huang, Tianqiong He, Zhijun Zhou, Jia Shen, Zhongdao Wu

**Affiliations:** 1 Zhongshan School of Medicine, Sun Yat-sen University, Guangzhou, 510080 Guangdong China; 2 Key Laboratory of Tropical Disease Control of the Ministry of Education, Sun Yat-sen University, Guangzhou, Guangdong China; 3 Department of Laboratory Animal Science, Xiangya School of Medicine, Central South University, Changsha, China; 4 Department of Zoology, Abdul Wali Khan University, Mardan, Pakistan; 5 Hainan General Hospital, Hainan Medical University, Haikou, China

**Keywords:** BRD4, Schistosomiasis, Immune response, Proinflammation

## Abstract

**Background:**

Schistosomiasis, a parasitic disease affecting more than 240 million people worldwide, is characterized by chronic inflammation and tissue fibrosis primarily induced by parasite egg deposition. Bromodomain-containing protein 4 (BRD4), an epigenetic and transcriptional regulator, has emerged as a potential therapeutic target due to its dual role in modulating *Schistosoma japonicum* (*S. japonicum*) reproductive development and organ fibrosis. Despite these advances, the specific involvement of BRD4 in the host immune response during *S. japonicum* infection still remains completely unclear.

**Methods:**

To explore the involvement of BRD4 in the immune response to *S. japonicum*, we performed a comparative time-series RNA-seq analysis of liver tissues from the non-permissive host *Microtus fortis* and the permissive host *Mus musculus*. BRD4-associated gene expression patterns were analyzed through correlation-based classification, followed by protein–protein interaction network construction and functional enrichment analyses. In addition, BRD4 was pharmacologically inhibited in vivo using JQ1, and hepatic inflammation and worm load were evaluated at 14 days post-infection.

**Results:**

BRD4 displayed distinct transcriptional dynamics between *M. fortis* and *M. musculus*. Genes positively correlated with BRD4 expression were significantly enriched in inflammatory and immune-related pathways, including Th17 cell differentiation and hallmark inflammatory response. These patterns suggest a potential regulatory role for BRD4 in mediating hepatic inflammation during infection. In vivo inhibition of BRD4 with JQ1 reduced liver inflammation, further supporting its association with proinflammatory responses.

**Conclusions:**

Our findings reveal strong transcriptional correlations between BRD4 expression and immune activation, and further highlight BRD4 as a potential regulator of host inflammatory responses during *S. japonicum* infection. BRD4 may serve as a valuable molecular target for understanding host–pathogen interactions and developing adjunct therapies against schistosomiasis.

**Graphic Abstract:**

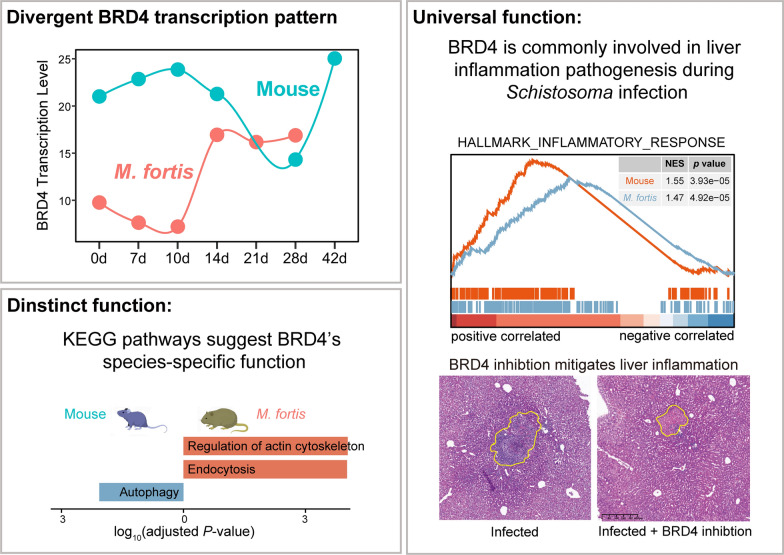

**Supplementary Information:**

The online version contains supplementary material available at 10.1186/s13071-025-06821-z.

## Background

Schistosomiasis, a widespread parasitic disease caused by trematode species of the genus *Schistosoma japonicum* (*S. japonicum*), continues to pose a significant global health threat, affecting approximately 240 million people, primarily in tropical and subtropical regions [1–4]. The main human-infecting species of schistosomes include *Schistosoma japonicum*, *Schistosoma mansoni*, and *Schistosoma haematobium*. In recent years, its geographical range has expanded, with increasing cases in urban areas and among travelers to endemic regions [5]. The disease is characterized by the deposition of parasite eggs within host tissues, leading to chronic inflammation and progressive hepatic fibrosis or other tissue fibrosis, a key pathological feature of schistosomiasis [6]. The host immune response to these eggs involves complex interactions between immune cells and the parasite antigen, which, if untreated, contribute to long-term tissue damage. Taking *S. japonicum* as an example to explain how schistosomes invade the human body, following skin penetration of the cercaria, schistosomula migrate through the bloodstream to portal-mesenteric venous system, where they mature sexually and begin egg deposition around 28 days post-infection (DPI) [2, 7]. At approximately 42 DPI, granulomatous inflammation develops in response to the eggs, triggering the activation of hepatic stellate cells (HSCs), a key step in liver fibrosis progression. In susceptible hosts, such as mice, this immune response results in chronic liver injury and fibrosis, particularly when the immune system fails to efficiently clear the parasite [8–10]. In contrast, *Microtus fortis* (*M. fortis*), the only known non-susceptible mammalian species to *S. japonicum*, prevents the parasite from maturing, leading to its extinction by 14 DPI. In this host, leukocyte adherence to the parasite peaks at 13 DPI, and parasite clearance is nearly complete by 21 DPI [11, 12]. Notably, after complete parasite elimination, liver inflammation in *M. fortis* resolves rapidly, with reports indicating substantial recovery by 42 DPI [13–15]. However, the immune and molecular mechanisms underlying these divergent responses remain poorly understood. Despite praziquantel being the primary therapeutic agent for schistosomiasis, its efficacy is limited by its inability to target immature schistosomes and mitigate egg-induced granulomatous inflammation, which contribute to fibrosis [16–18]. Furthermore, growing concerns over emerging drug resistance threaten the long-term efficacy of praziquantel as the sole treatment option. These challenges highlight the need for alternative therapeutic strategies that target both the parasite’s life cycle and the host’s inflammatory responses. Understanding the molecular mechanisms underlying host resistance to *S. japonicum* is crucial for developing novel therapies.

Recent studies highlight BRD4, a broad and essential epigenetic and transcriptional regulator, as a promising target in fibrosis and immune modulation. As an acetylation reader and a transcriptional co-activator, BRD4 regulates immune gene expression and inflammatory responses to pathogens [19]. Notably, BRD4 has been implicated in HSCs activation via the JAK2/STAT3 signaling pathway, a crucial driver of liver fibrosis [20–22]. Inhibition of BRD4 with JQ1 has been shown to reduce hepatic granulomas in *S. japonicum*-infected mice by impairing parasite reproductive development and egg production [23]. Although BRD4’s role in hepatic fibrosis progression and its effects on *S. japonicum* have been explored, whether it contributes to host resistance to *S. japonicum*, and its potential role in mediating immune response to *S. japonicum*, remains poorly understood.

This study aims to bridge this gap by investigating BRD4’s potential role in regulating the immune response to *S. japonicum* infection. By analyzing comprehensive time-course RNA-seq datasets from *M. fortis* and murine models at various post-infection intervals, we characterized the dynamic transcription patterns of BRD4. Additionally, we validated BRD4’s proinflammatory role in vivo through the administration of JQ1. These findings contribute to the understanding of BRD4’s broader immunological significance, emphasizing it as a potential target for its comprehensive regulatory role in host responses to *S. japonicum* infection and *S. japonicum* reproductive development and fibrosis pathogenesis. Thus, this study provides a foundation for developing novel anti-parasitic therapeutic strategies.

## Methods

### Parasites and animals

Cercariae of *S. japonicum* were sourced from infected *Oncomelania hupensis* snails, which were procured from the Institute of Parasitic Diseases affiliated with the China CDC, located in Shanghai. Adult male BALB/c mice, aged 6–8 weeks, were obtained from the Medical Experimental Animal Center in Guangdong Province, Guangzhou, China. Additionally, *M. fortis* were provided by the Animal Health Center at Xiangya School of Medicine, Central South University, Hunan, China. All animal experiments were conducted in strict accordance with the recommendations of the Laboratory of Animal Welfare and Ethics Committee (LAWEC) of China. Animals were euthanized by cervical dislocation under anesthesia to minimize suffering.

### Intraperitoneal injection

JQ1 (HY-13030, MedChemExpress, USA) was reconstituted in a 20% solution of hydroxypropyl-β-cyclodextrin (HP-β-CD, HY-101103, MedChemExpress, USA) in saline to a final concentration of 10 mg/mL. *M. fortis* were intraperitoneally administered JQ1 at a dose of 50 mg/kg body weight, beginning 7 DPI and continuing once daily for 7 days. The control group received an equivalent volume of vehicle solution, consisting of 10% (wt/vol) HP-β-CD. Animals were euthanized 24 h after the final injection. Liver tissues were collected for subsequent experimental analyses.

### Animal infection and sample collections

In accordance with the established susceptibility profiles of rodent species to *S. japonicum*, each BALB/c mouse was subjected to percutaneous infection with 40 cercariae of *S. japonicum*, while *M. fortis* were exposed percutaneously to a higher dose of 200 cercariae. This differential dosing strategy was employed to account for the varying infection dynamics and host–parasite interactions between the two rodent species. Following the designated infection periods, adult worms were retrieved through perfusion of the portal venous system, a method optimized to maximize parasite recovery while minimizing tissue damage. The harvested worms were meticulously counted and imaged using a stereoscopic microscope (M205FA, Leica, Germany) to document their morphological features and quantify parasite burden. Concurrently, the livers of infected animals were excised and processed for histopathological examination to assess the extent of *S. japonicum*-induced pathology.

### Time-course RNA-seq data analysis

Time-course RNA-seq data were preprocessed using RSEM (version 1.2.28) to generate raw read counts and transcript-level quantifications. The reference transcriptome was prepared using the rsem-prepare-reference function with the parameters –bowtie2 -p 25, which enabled efficient indexing for subsequent read alignment. Raw read counts and transcript per million (TPM) values were then obtained using the rsem-calculate-expression function, configured with the parameters –paired-end –bowtie2 –bowtie2-k 20 –output-genome-bam –sort-bam-by-coordinate. These settings ensured accurate quantification of gene and transcript expression levels while accommodating paired-end sequencing data. The Bowtie2 alignment parameters were optimized for sensitivity and accuracy, with the following settings: -q –sensitive –dpad 0 –gbar 99,999,999 –mp 1,1 –np 1 –score-min L,0,−0.1 -I 1 -X 1000 –no-mixed –no-discordant -p 8 -k 20 -N 1. To normalize the raw read counts, the DESeq2 package was employed. This normalization step is crucial for minimizing systematic variations and ensuring that gene expression levels can be accurately compared across samples. The normalized data were subsequently used for downstream analyses to identify differentially expressed genes and pathways.

### Mfuzz clustering analysis

Gene clusters were obtained using Mfuzz package in R with fuzzy c-means algorithm. The optimal number of clusters (c) and the fuzziness parameter (m) were determined through the partition coefficient analysis, which evaluates the uniformity of clustering. The clustering results were visualized using the mfuzz.plot function, with each cluster representing a distinct temporal expression pattern. The membership values of genes in each cluster were color-coded to indicate their degree of association with the cluster.

### Pathway enrichment analysis

Pathway enrichment analysis was conducted using the clusterProfiler[24] package (version 4.10.0) in R (version 4.3.0) to identify significantly enriched biological pathways. The analysis was performed using both the enrichGO and enrichKEGG functions. The adjusted *P*-value cutoff was set at 0.05, and the Benjamini–Hochberg method was used for multiple testing correction. Significant pathways were identified on the basis of the adjusted *P*-values, ensuring that only pathways with a stringent statistical significance were reported.

### Protein–protein interaction network

Protein–protein interaction network was constructed using STRING database. The interactions were filtered with a confidence score threshold of 0.4 to ensure reliability. The network was subsequently visualized and analyzed using Cytoscape (version 3.10.3). Nodes in the network represent individual proteins, while edges denote interactions between them.

### Multiple sequence alignment

Protein sequences were retrieved from the UniProt database and the NCBI RefSeq genome assembly (GCF_014885135.2). Multiple sequence alignment was performed using the alignment function provided by UniProt, which facilitates accurate and efficient alignment of protein sequences. The aligned sequences were subsequently visualized using Jalview (version 2.11.4.1), a versatile tool for analyzing and displaying sequence alignments. Sequence conservation and variability were highlighted using the Clustal color scheme.

### Gene set enrichment analysis (GSEA)

Gene set enrichment analysis (GSEA) was conducted using the R programming environment, leveraging the GSEA, gseGO, and gseaKEGG functions from the clusterProfiler package. For this analysis, hallmark gene sets derived from the Mouse Signature Database (MSigDB) and functional annotations of both mouse and *M. fortis* were utilized. The statistical significance of GSEA was assessed using a false discovery rate (FDR) threshold of 0.25 and an absolute normalized enrichment score (NES) exceeding 1. Genes were ranked according to their expression correlation with BRD4. The results were visualized using the enrichplot package, which provides a comprehensive graphical representation of the analysis. To estimate empirical *P*-values for the gene sets, a total of 1000 permutations were performed.

### In vitro schistosomulum killing assays

Macrophages and schistosomula were co-cultured at an effector-to-target ratio of (1–2) × 10^3^:1 in RPMI-1640 medium supplemented with 10% heat-inactivated fetal bovine serum (FBS), penicillin (100 U/mL), and streptomycin (100 mg/mL). As we have reported in another work, macrophages are involved in attaching and killing schistosomula [12]. Notably, for the initial comparison of schistosomulum-killing activities after JQ1 administration, non-heat-inactivated FBS was utilized. Throughout these experiments, no direct cytotoxic effects of the reagents on schistosomula were observed at the concentrations employed. Co-cultures were incubated for 20 h at 37 °C in an atmosphere of 5% CO₂. Schistosomulum killing ratios were assessed at 6 h and 20 h after incubation with JQ1 (2 μM) or not.

### Histopathology

Liver tissues were initially subjected to fixation in a 4% paraformaldehyde (PFA) solution, followed by embedding in paraffin wax to facilitate histological processing. Subsequently, ultrathin sections were generated using an ultramicrotome and were meticulously collected on glass slides. After a comprehensive dewaxing procedure to eliminate residual paraffin, the sections underwent hematoxylin and eosin (H&E) staining to elucidate the intricate cellular and tissue architecture. The stained sections were then imaged at a magnification of 10× using an automated slide scanning system (AxioScan.Z1, Zeiss, Germany), thereby enabling high-resolution morphological analysis.

### Scanning electron microscopy

Scanning electron microscopy (SEM) was performed as previously described [25]. Worms were fixed in 2.5% glutaraldehyde prepared in 0.2 M PBS (pH 7.4) at 4 °C for over 24 h. Fixed samples were dehydrated through a graded ethanol series (50–100%, v/v), followed by sequential treatment with acetone and isoamyl acetate. After critical point drying, samples were coated with gold to enhance conductivity. SEM imaging was conducted using either a FEl Quanta 200 (FEI, USA) or a Zeiss Crossbeam 550 (Zeiss, Germany). Morphometric analysis of worm size was performed using ImageJ software, which can calculate the absolute area of worms in the image on the basis of the scale.

### RNA extraction and quantitative real-time PCR

Total RNA was isolated from liver tissues employing TRIzol, followed by purification with the RNeasy Mini Kit (Qiagen, USA), according to the manufacturer’s instructions, as previously described [26]. cDNA synthesis and quantitative real-time PCR were conducted using the SYBR Green Master Mix (Takara, Japan) on the CFX96 Real-Time PCR Detection System (Bio-Rad, USA). Relative gene expression was determined by normalizing to GAPDH and calculated using the 2^−ΔΔCT^ method. Primer sequences are listed in Additional file 1: Table S1.

### Statistical analysis

All graphical representations and statistical analyses were conducted using the R programming environment. Data were analyzed using Student’s unpaired *t*-test with Welch’s correction to account for potential differences in variance between two groups. Pearson correlation analysis was employed to assess the strength and direction of linear relationships between variables. A *P*-value threshold of less than 0.05 was established as the criterion for statistical significance. ns: not significant, *: *P* < 0.05, **: *P* < 0.01, ***: *P* < 0.001, ****: *P* < 0.0001.

## Results

### Temporal changes in BRD4 transcription are related to hepatic pathological changes

**Fig 1:**
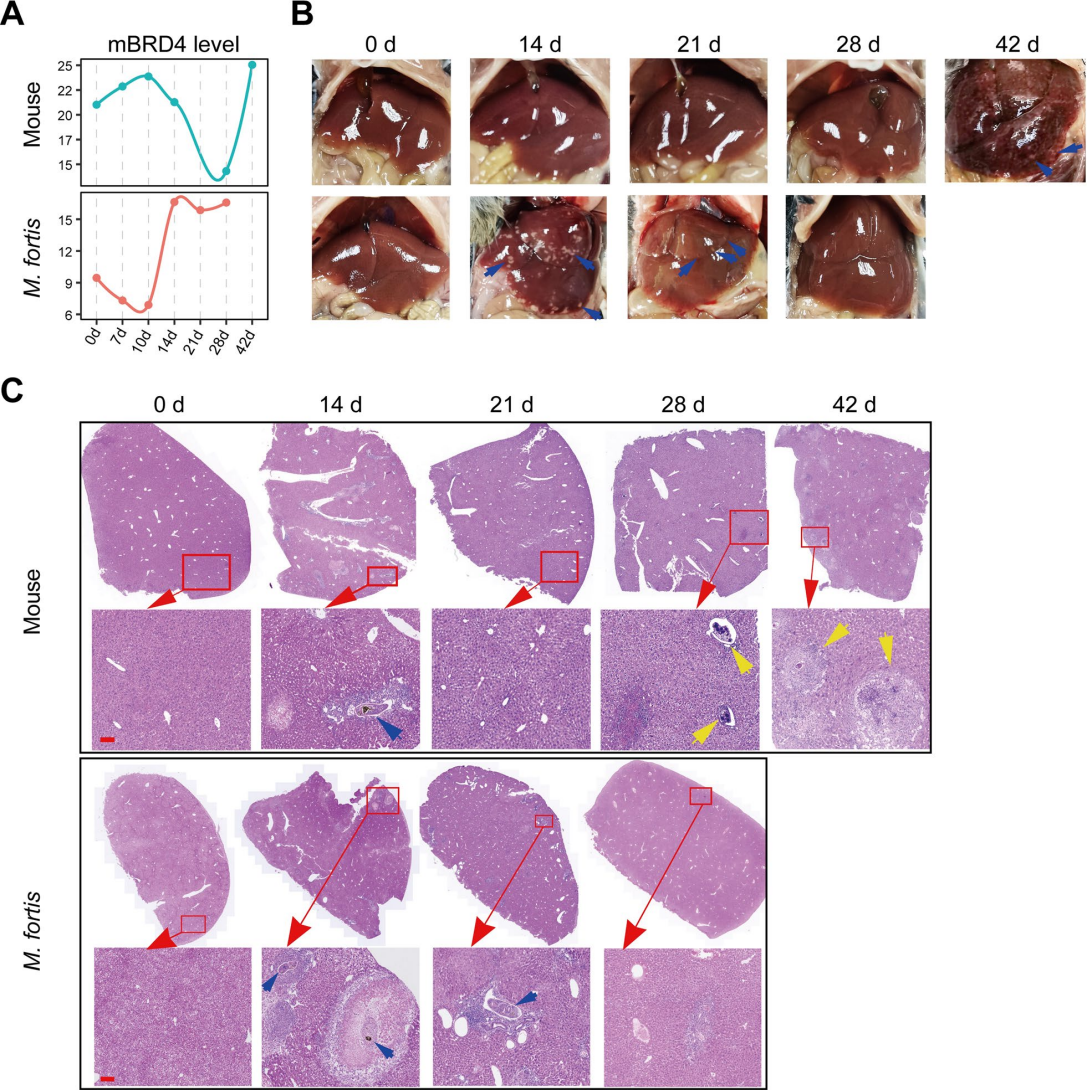
Temporal analysis of BRD4 transcription and hepatic pathology in *M. fortis* and mice during *S. japonicum* infection. **A** Temporal dynamics of BRD4 transcription in the liver of *M. fortis* and mice. The transcriptional level is normalized to transcripts per million (TPM). Three replicates for each organism at each timepoint. **B, C** Histopathology of the liver of mice and *M. fortis* infected with *S. japonicum* at the indicated days post-infection. **B** Gross pathology of the liver. Blue arrows indicate the white nodules. **C** Representative H&E staining images of the liver. Blue arrows indicate the worm, the yellow arrows indicate the egg-granulomas. The experiment was repeated twice with three biological replicates; scale bars represent 100 μm

To ascertain BRD4’s involvement in the immune response against *S. japonicum*, we initially investigated its transcriptional dynamics in the livers of *M. fortis* and mice using time-series RNA-seq datasets obtained from Gene Expression Omnibus (GEO) database. The temporal transcriptional profiles of BRD4 exhibit divergent patterns between *M. fortis* and mice (Fig. 1A). In *M. fortis*, the BRD4 transcription level increases at 14 DPI and persists at an elevated state thereafter. Conversely, in mice, as infection progresses, the BRD4 transcription level begins to decline around 10 DPI and continues to decrease until approximately 28 DPI. Notably, by 42 DPI, it shows a significant increase. Interestingly, the transcriptional profiles of BRD4 correlate with the pathological phenotypes observed in *M. fortis* and mice. White inflammatory nodules are evident on the liver surface of *M. fortis* at 14 DPI and in mice at 42 DPI, coinciding with the elevated transcription levels of BRD4 (Fig. 1B). Histopathological observations reveal that inflammatory cell infiltration occurs at 14 DPI in *M. fortis* and at 42 DPI in mice (Fig. 1C), indicative of the immune response to the parasite. Together, these findings suggest that BRD4 might play important roles in the immune process against *S. japonicum*. Recent studies have increasingly implicated BRD4 in the regulation of non-alcoholic steatohepatitis, liver fibrosis, and inflammation [27, 28], thereby reinforcing its potential role in immune responses to *S. japonicum* infection.

### BRD4 acts as a central regulatory hub in the functionally conserved network shared by mouse and *M. fortis*

**Fig 2:**
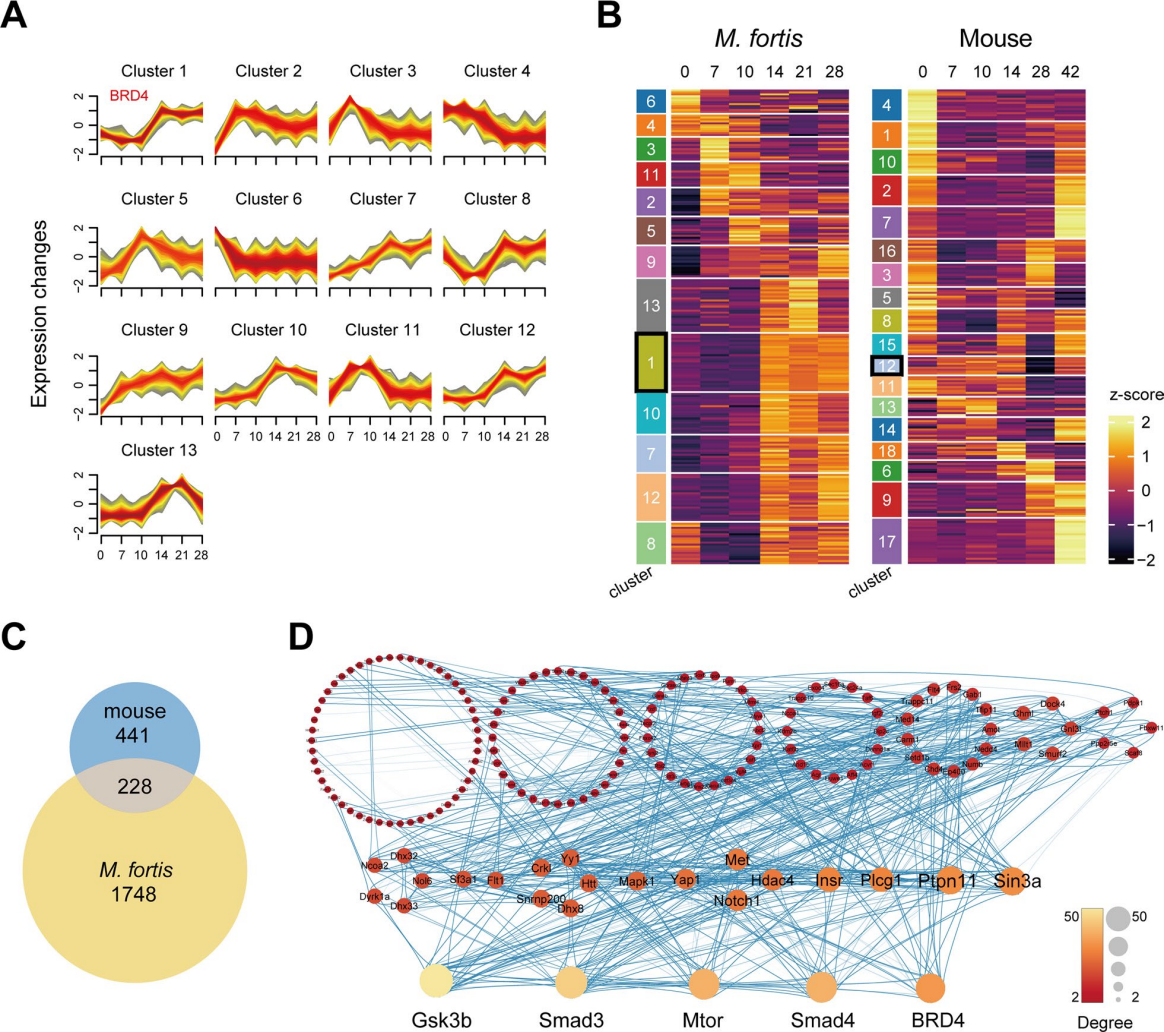
Comparative analysis of gene transcriptional profile and protein interaction networks in *M. fortis* and mice post-infection. **A** Mfuzz clustering analysis of genes in *M. fortis*. Red-colored lines denote genes with high membership values, whereas yellow-colored lines highlight genes with low membership values. The *x*-axis represents days post-infection. **B** Heatmap showing gene transcription level in *M. fortis* and mice. Clusters correspond to the Mfuzz analysis result. **C** Venn diagram depicts the overlap between cluster 1 in *M. fortis* and cluster 12 in mice, which have similar transcriptional pattern as BRD4. **D** Protein–protein interaction network of the shared 228 gene indicates the leading role of BRD4. Node size and color correspond to the degree of connectivity

To further elucidate the potential role of BRD4 in the anti-parasitic process, we employed the fuzzy c-means clustering algorithm to categorize genes with transcriptional profiles congruent with that of BRD4 throughout the time course of infection. This analysis yielded 1976 genes in cluster 1 of *M. fortis* and 669 genes in cluster 12 of mice, both exhibiting expression patterns analogous to BRD4 (Fig. 2A, B; Additional file 1: Fig. S1). Given the association of BRD4 with histopathological manifestations in both species and the high degree of conservation of its protein sequence, particularly within the functional domains (Additional file 1: Fig. S2, S3), we hypothesize that BRD4 may fulfill analogous roles in these species. Consequently, we focused on the 228 genes that intersected between the two BRD4-like gene clusters (Fig. 2C). A protein–protein interaction network analysis further revealed that BRD4 functions as a central regulatory hub within this shared gene set (Fig. 2D). This result aligns with previous studies indicating that BRD4 functions as critical regulator in controlling gene expression of IBD-associated inflammatory cytokine networks [29]. It is also consistent with the characteristics of BRD4 as a well-known epigenetic reader and transcriptional regulator [30, 31]. These findings suggest that BRD4 may orchestrate a conserved response to *S. japonicum* across species.

### BRD4 universally regulates inflammation-associated pathways in *M. fortis* and mice

**Fig 3:**
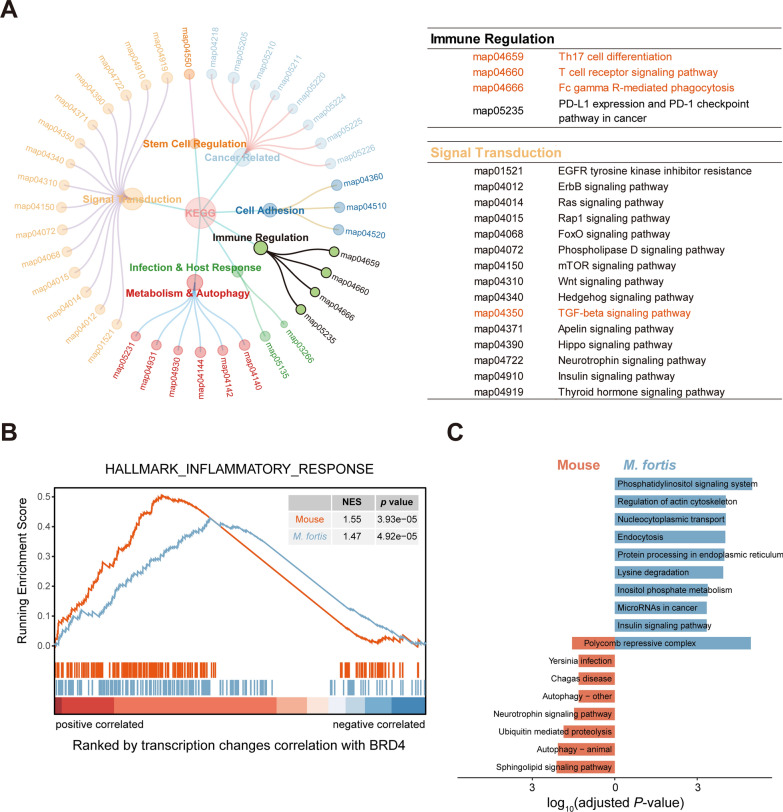
BRD4 functional characterization. **A** Kyoto Encyclopedia of Genes and Genomes (KEGG) pathway enrichment analysis of the 228 intersection genes. Map ID corresponds to the pathway in KEGG database, all the pathways shown are significantly enriched. **B** GSEA results for the Hallmark Inflammatory Response gene set in mouse and *M. fortis*. Genes were ranked on the basis of their transcription changes correlation with BRD4 during the infection process. Red: mouse; Blue: *M. fortis*. **C** Top 10 enriched KEGG pathways of the *M. fortis*-specific BRD4-like genes, and the enriched KEGG pathways of the mouse-specific BRD4-like genes

Having identified 228 genes potentially under the regulatory influence of BRD4, we proceeded to conduct a functional enrichment analysis aimed at exploring the potential biological functions of BRD4. The analysis revealed a significant enrichment of pathways related to signal transduction and immune regulation. Among these, several pathways were implicated in immune responses, including Th17 cell differentiation, TGF-beta signaling pathway, T cell receptor signaling pathway, and Fc gamma R-mediated phagocytosis (Fig. 3A). The link of BRD4 and these pathways were also reported in previous studies [32–35]. Given the intrinsic link between immune regulatory pathways and inflammatory processes, we performed a Gene Set Enrichment Analysis (GSEA) by ranking genes on the basis of their expression correlation with BRD4 within the context of the hallmark inflammatory response gene set. The GSEA findings revealed a positive correlation between most inflammation-related genes and BRD4 at the transcriptional level during *S. japonicum* infection (Fig. 3B, Additional file 2: Table S2). Additionally, genes that exhibited positive correlation with BRD4 were significantly enriched in the biological process of positive regulation of acute inflammatory responses (Additional file 2: Table S3). This finding is in line with previous studies demonstrating that BRD4 exerts proinflammatory effects through regulating a variety of immune and inflammatory genes [19]. These findings suggest that BRD4 may play a pivotal role in modulating proinflammatory processes in response to *S. japonicum* infection within the liver of *M. fortis* and mice.

We also compared the functional differences of the two species-specific BRD4-like genes. The results show that *M. fortis*-specific BRD4-like genes are primarily associated with pathways such as endocytosis and regulation of actin cytoskeleton, whereas mouse-specific BRD4-like genes are significantly enriched in the autophagy pathway (Fig. 3C, Additional file 2: Table S4). These results suggest that BRD4 might contribute to the cellular endocytic processes and cytoskeletal remodeling in *M. fortis*.

### Blocking the function of BRD4 reduced the inflammatory infiltration

**Fig 4:**
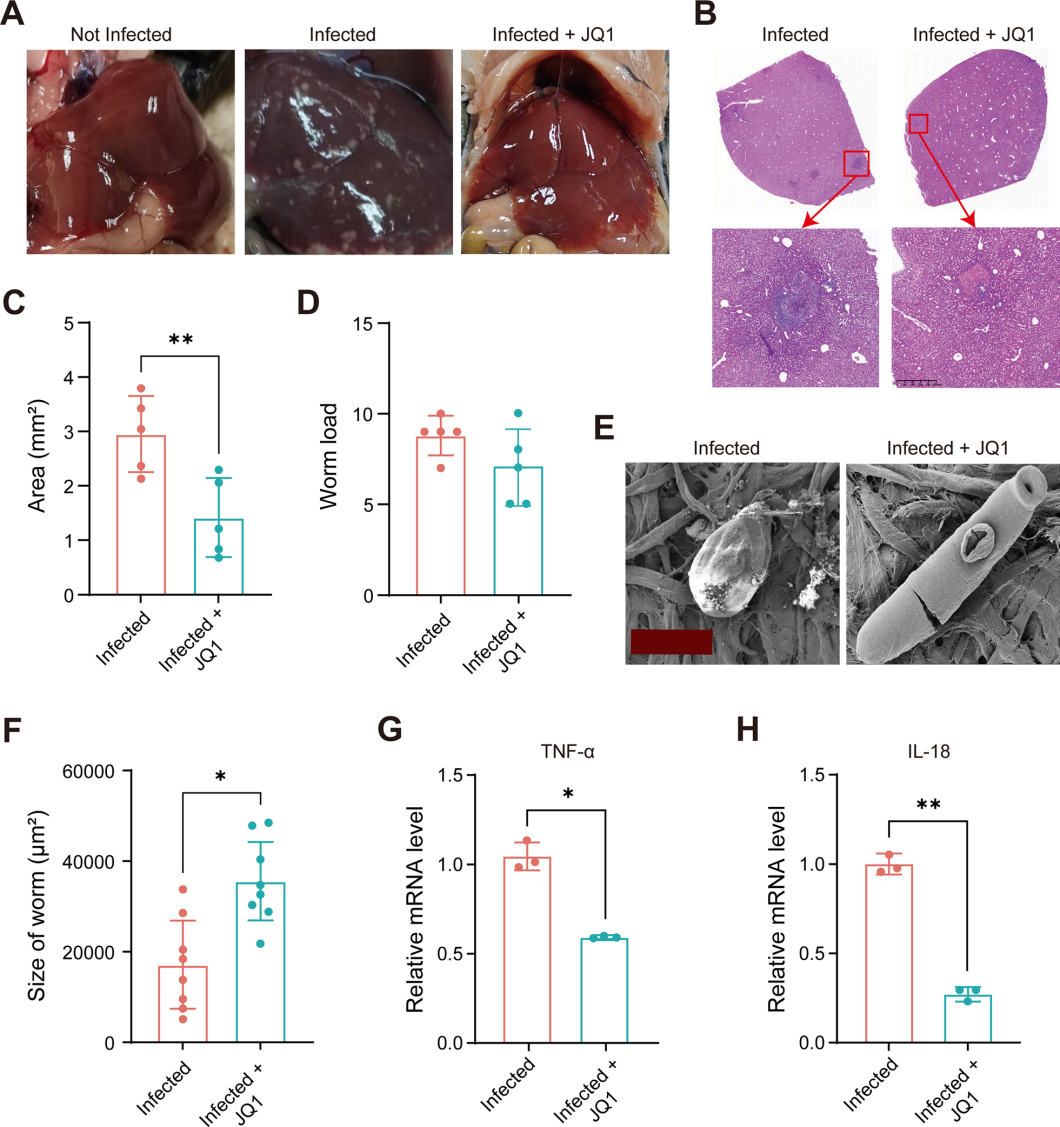
Functional validation in vivo.** A** Gross pathology of the *M. fortis* liver in 14 DPI. **B** Representative H&E staining images of the liver of *M. fortis* in 14 DPI. Scare bars represent 400 μm. **C** Statistical analysis of the area of inflammatory infiltration. **D** *S. japonicum* number in liver after JQ1 treatment in 14 DPI. **E**, **F** The development of *S. japonicum* in liver after JQ1 treatment in 14 DPI. Scare bars represent 100 μm. Representative image shown. The experiment was repeated twice with three biological replicates. **G**, **H** Effect of JQ1 treatment on mRNA expression of proinflammatory cytokines. RT–qPCR was used to measure TNF-α and IL-18 mRNA level in liver tissue, three replicates per condition. Data are presented as mean ± standard deviation. *: *P* < 0.05; **: *P* < 0.01 as determined by unpaired student’s *t*-test

To experimentally validate the role of BRD4 in promoting inflammatory infiltration, we treated infected *M. fortis* with JQ1, an inhibitor which impairs BRD4 function by competitively binding to its bromodomains. This binding prevents BRD4 from recognizing acetylated histones, thereby displacing it from chromatin and ultimately disrupting its role as an epigenetic reader and transcriptional regulator. Treatment commenced at 7 DPI and was administered daily via intraperitoneal injection until 14 DPI. Our observations revealed a marked reduction in both the number and size of white nodules on the liver surface, as well as a decrease in inflammatory infiltration following JQ1 treatment (Fig. 4A–C). This result is consistent with previous reports that JQ1 alleviates hepatic inflammation in *Listeria monocytogenes*- and *S. japonicum*-infected mice [36, 37]. However, no significant impact of JQ1 on the survival of *S. japonicum* was observed in vivo (Fig. 4D) and in vitro (Additional file 1: Fig. S4) in this study, but the size of worm increased in JQ1 administrated group (Fig. 4E, F; Additional file 1: Material S1). This increase might be attributed to the reduced inflammatory infiltration, which in turn leads to a decreased attack on the worm. We also measured the mRNA expression levels of proinflammatory cytokines after JQ1 treatment. The results showed a notable decrease in the expression of tumor necrosis factor (TNF)-α and interleukin (IL)−18, both of which play important roles in mediating inflammatory responses (Fig. 4G, H), and the reduction of IL-18 after JQ1 treatment is also reported in previous studies[38]. Collectively, these findings suggest that BRD4 plays an important role in promoting inflammatory infiltration, thereby corroborating the insights derived from our functional enrichment analysis and GSEA.

## Discussion

In this study, we demonstrate that BRD4 exhibits divergent temporal transcriptional patterns in the non-permissive host (*M. fortis*) and the permissive host (mice) during *S. japonicum* infection. Functional analysis and protein–protein interaction networks suggested that BRD4 may serve as a central regulatory node, modulating multiple biological processes, particular inflammation-related pathways in both hosts. In vivo assays indicated that BRD4 inhibition reduced the inflammatory infiltration.

Contrary to previous reports suggesting that BRD4 affects pathogen clearance in bacterial infections [39], our study found no significant effect of BRD4 inhibition on *S. japonicum* clearance ability in *M. fortis*. Firstly, expanding the sample size is essential to further validate the impact of BRD4 inhibition on *S. japonicum* survival. A more effective experimental system, such as the conditional knockout of BRD4 in the liver of *M. fortis* and mice, is necessary to distinguish the direct effects of BRD4 inhibition on the parasite from those mediated by the host, thereby enabling a more comprehensive dissection of BRD4’s effect on *S. japonicum*. Secondly, this discrepancy could be attributed to fundamental differences in the immune mechanisms governing bacterial versus parasitic infections.

Additionally, our findings align with prior studies suggesting that BRD4 acts as a key regulator of immune pathways across various infectious and inflammatory diseases [19, 23, 37], including bacterial [40, 41] and viral infections [42, 43]. BRD4 has been identified as a potential therapeutic target for fibrosis resulting from chronic inflammation, through its involvement in hepatic stellate cell activation and hepatic fibrosis development via the P300/H3K27ac/PLK1 axis [41] or the NF-kB pathway [44], as well as its role in mitigating *S. japonicum* granulomas resulting from egg deposition [23]. Here, we found that BRD4 may regulate immune gene expression associated with Th17 differentiation, TGF-β signaling, and Fc gamma R-mediated phagocytosis, which collectively drive inflammatory responses in both susceptible and non-susceptible hosts. These results align with previous studies. Li Y and Cheung KL et al. have reported that BRD4 positively regulate mouse Th17 cell differentiation, and its inhibition dramatically reduced Th17 cell activity [32, 45]. Chen M et al. reported the regulatory loop of BRD4/STAT3 and TGF-β [34]. Banham GD et al. reported that BRD4 inhibition affect genes relating to FcγR-mediated phagocytosis pathway [35]. Our study extends this understanding by highlighting BRD4’s role in parasitic infections such as schistosomiasis, which involve distinct immune regulatory mechanisms compared with bacterial or viral infections.

The findings that BRD4 may contribute to the cellular endocytic processes and cytoskeletal remodeling in *M. fortis* also are consistent with previous studies. Recently, trogocytosis-mediated killing of the unicellular parasite *Trichomonas vaginalis* and *S. japonicum* have been identified [12, 46]. Trogocytosis, which describes the phenomenon of one cell acquiring part of the plasma membrane and cytoplasm of another cell through direct contact, is contact-dependent and requires actin cytoskeletal rearrangement [47]. Bigger-Allen A et al. reported that JQ1 was able to affect cytoskeletal rearrangement [48]. This mechanistic link suggests that BRD4 might play roles in cytoskeletal remodeling, thereby enhancing immune surveillance and facilitating rapid parasite clearance in *M. fortis*. Conversely, the enrichment of autophagy-related pathways in mice appears discordant, with existing studies demonstrating that autophagy is inhibited at 2, 4, and 6 weeks post-infection with *S. japonicum *[49]. This discrepancy may arise from compensatory mechanisms within the BRD4-mediated autophagy regulatory network, which counteract the inhibitory effects imposed by *S. japonicum* infection. These results may explain the different outcomes of infection between *M. fortis* and mice in some degrees. Furthermore, the functional annotation of the *M. fortis* still lags that of mice, highlighting the need for continued research efforts to bridge the gap and facilitate a better dissection of the species-specific immune differences between *M. fortis* and mice.

JQ1, a small molecule inhibitor of BRD4, has shown promise in preclinical models of inflammatory diseases and cancers. This study extends these findings to schistosomiasis by emphasizing its involvement in the host immune response to *S. japonicum*. Together, this study declares BRD4 an attractive candidate for combination therapies, particularly in conjunction with praziquantel, to clear *S. japonicum*, ameliorate *S. japonicum*’s reproductive development, and reduce host inflammatory response to *S. japonicum* simultaneously.

While this study provides a valuable foundation for understanding the role of BRD4 in schistosomiasis, several questions remain unanswered. The precise molecular mechanisms through which BRD4 modulates immune pathways in the context of schistosomiasis are still not fully understood. Further mechanistic studies, including Cleavage Under Targets and Tagmentation (CUT&Tag) assays, gene perturbation experiments and single-cell RNA sequencing, are necessary to delineate the core regulatory axis governing BRD4-mediated inflammation and to identify the primary cell types responsible for the different response against parasites. The precise mechanisms behind the temporal dynamics of BRD4’s up-regulation at 14 DPI in *M. fortis* versus 42 DPI in mice, and its down-regulation during worm maturation in mice, also remain confused. Advanced methodologies, such as deep learning (DL), which could potentially provide valuable insights by uncovering novel regulatory networks and key factors group involved in the host–pathogen interactions in schistosomiasis, should be used in the future investigation. Moreover, such approaches may also help elucidate the mechanisms underlying the innate capacity to clear *S. japonicum* in *M. fortis*. Additionally, the potential for combining BRD4 inhibition with current treatments such as praziquantel warrants further investigation. Clinical studies are needed to evaluate the efficacy and safety of such combination therapies, particularly in endemic areas where schistosomiasis remains a persistent health threat. Understanding the potential synergy between BRD4 inhibitors and other therapeutic agents could pave the way for more effective treatment strategies.

## Conclusions

This study identifies BRD4 as a potential regulator of host immune responses to *S. japonicum* infection, particularly in the context of inflammation. Moreover, this research lays the groundwork for combining BRD4-targeted therapies, such as JQ1, with conventional treatments such as praziquantel, which could enhance therapeutic efficacy and simultaneously mitigate severe liver fibrosis and other complications associated with chronic schistosomiasis and other parasitic diseases. In this study, we comprehensively identified the gene transcriptional patterns during *S. japonicum* infection in both *M. fortis* and mice. It may provide valuable insights for future research into the molecular mechanism underlying natural host resistance to *S. japonicum*, and may inform strategies for conferring resistance to susceptible host.

## Supplementary Information

Additional file 1. Table S1. Sequences for primers used in RT-qPCR. Figure S1. Mfuzz clustering analysis result of mice. Figure S2. Protein sequence alignment result of BRD4 between mouse and *M. fortis*. Figure S3. BRD4 percent identity between *Homo sapiens*, *Mus musculus* and *M. fortis.* Figure S4. In vitro schistosomulum killing assay. Material S1. Images of scanning electron microscopy.

Additional file 2. Table S2. GSEA of HALLMARK_INFLAMMATORY_RESPONSE gene sets. Table S3. GSEA of gene sets in GO and KEGG terms. Table S4. GO and KEGG enrichment analysis of *M. fortis*-specific, mouse-specific, shared BRD4-like genes.

## Data Availability

This study did't generate new sequencing datasets. All RNA-seq data analyzed inthis study were obtained from the GEO database under GSE101654 and GSE101656. All materials supporting the conclusions of this study are available within the article and its Supplementary Information files.

## Abbreviations

BRD4: Bromodomain-containing protein 4
DPI: Days post-infection
GSEA: Gene set enrichment analysis
HSCs: Hepatic stellate cells
KEGG: Kyoto Encyclopedia of Genes and Genomes
*M. fortis*: *Microtus fortis*
