# Supplementary material for: Comparative analysis of Microtus fortis and murine hosts reveals a correlation between BRD4 and hepatic inflammation during Schistosoma japonicum infection
Source: Parasit Vectors. 2025 Jul 4;18:259. doi: 10.1186/s13071-025-06821-z (PMC12228314; doi:10.1186/s13071-025-06821-z)
Supplement: Supplementary file 1 — Additional file 1. Table S1. Sequences for primers used in RT-qPCR. Figure S1. Mfuzz clustering analysis result of mice. Figure S2. Protein sequence alignment result of BRD4 between mouse and M. fortis. Figure S3. BRD4 percent identity between Homo sapiens, Mus musculus and M. fortis. Figure S4. In vitro schistosomulum killing assay. Material S1. Images of scanning electron microscopy. [file 13071_2025_6821_MOESM1_ESM.docx]

# Supplementary materials for

Comparative analysis of *Microtus fortis* and murine hosts reveals a correlation between BRD4 and hepatic inflammation during *Schistosoma* *japonicum* infection

**Ming Yuan^1,2†^, Mingrou Wu****^1,2†^, Yunyi Hu^1,2^, Siyu Zhao^1,2^, Jehangir Khan^4,5^, Zhanhong Yuan^1,2^, Yun Huang^1,2^, Tianqiong He^3^, Zhijun Zhou^3*^, Jia Shen^1,2*^****, Zhongdao Wu^1,2*^**

_1_ Zhongshan School of Medicine, Sun Yat-sen University, Guangzhou, Guangdong 510080, China.

_2_ Key Laboratory of Tropical Disease Control of the Ministry of Education, Sun Yat-sen University, Guangzhou, Guangdong, China.

_3_ Department of Laboratory Animal Science, Xiangya School of Medicine, Central South University.

_4_ Department of Zoology, Abdul Wali Khan University, Mardan, Pakistan.

_5_ Hainan General Hospital, Hainan Medical University, Haikou, China.

† These authors contributed equally to this work.

* Correspondence: Zhijun Zhou ([zhouzhijun@csu.edu.cn](mailto:zhouzhijun@csu.edu.cn)) or Jia Shen ([shenj29@mail.sysu.edu.cn](mailto:shenj29@mail.sysu.edu.cn)) or Zhongdao Wu ([wuzhd@mail.sysu.edu.cn](mailto:wuzhd@mail.sysu.edu.cn)).

Table S1 Sequences for primers used in RT-qPCR. GAPDH, glyceraldehyde 3-phosphate dehydrogenase; TNF-α, tumor necrosis factor alpha; IL-18, interleukin 18.

| Gene | Primer | Sequence (5’-3’) |
| --- | --- | --- |
| GAPDH | Forward  Reverse | CCACCCATGGCAAGTTCAAA  ATCTCGCTCCTGGAAGATGG |
| TNF-α | Forward  Reverse | GGTGCCTATGTCTCAGCCTCTT  GCCATAGAACTGATGAGAGGGAG |
| IL-18 | Forward  Reverse | GAAGGCCACCTGTGTTTGAG  TCATGCTTCACAGAGAGGGT |


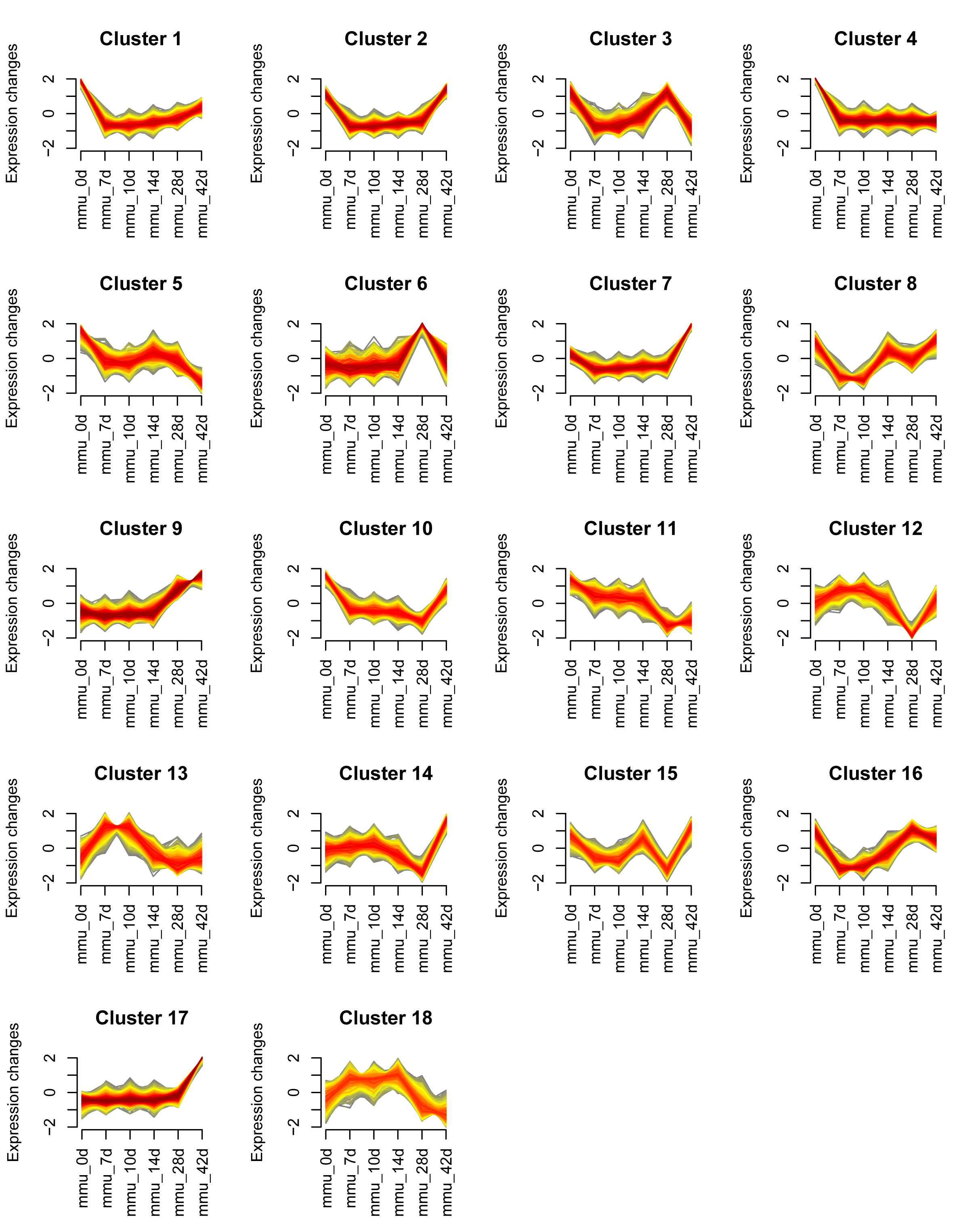


Fig. S1 Mfuzz clustering analysis result of mice. Red-colored lines denote genes with high membership values, whereas yellow-colored lines highlight genes with low membership values. The x-axis represents days post-infection.

Fig. S2 Protein sequence alignment result of BRD4 between mouse and *M. fortis*. The dark brown block represents the bromodomain BD1, blue block represents the bromodomain BD2 and light blue block represents the C-terminal domain of BRD4 in mouse. Conservation was auto calculated by Jalview.


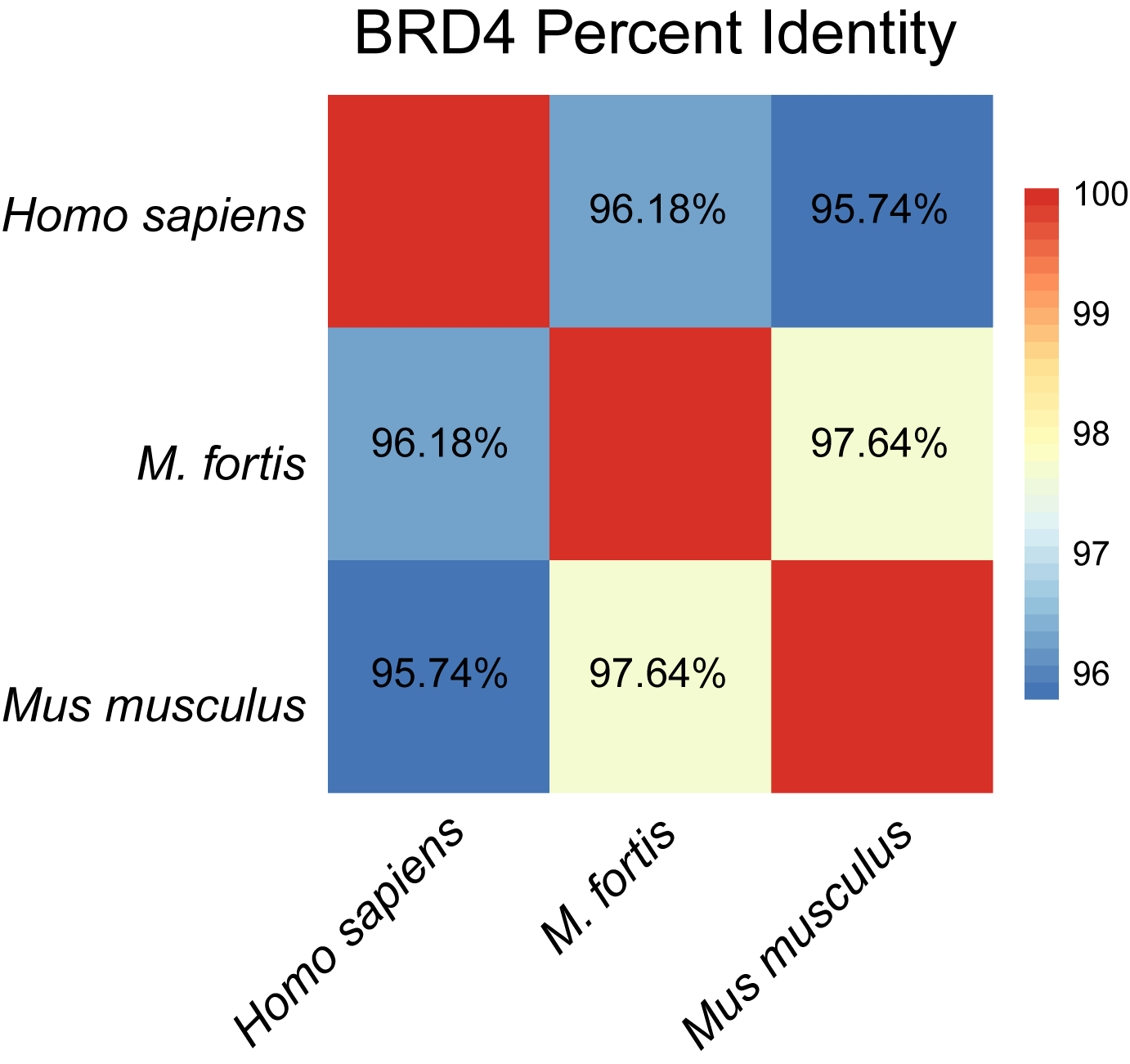


Fig. S3 BRD4 percent identity between *Homo sapiens*, *Mus musculus* and *M. fortis*.


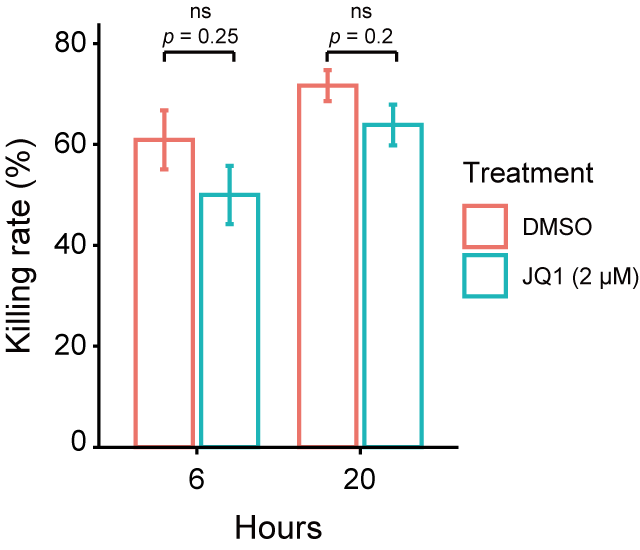


Fig. S4 In vitro schistosomulum killing assay. As we have reported that macrophages are involved in attaching and killing schistosomula in another work[12], macrophages and schistosomula were co-cultured with 2 μM JQ1 treatment (blue) or not (red). The schistosomula killing rate was assessed at 6, 20 hours after incubation. The experiment was repeated twice with three biological replicates. Student's t-test was used for statistics. ns: not significant.

Material S1 Images of scanning electron microscopy.

| Infected Group: |  |  |
| --- | --- | --- |
| 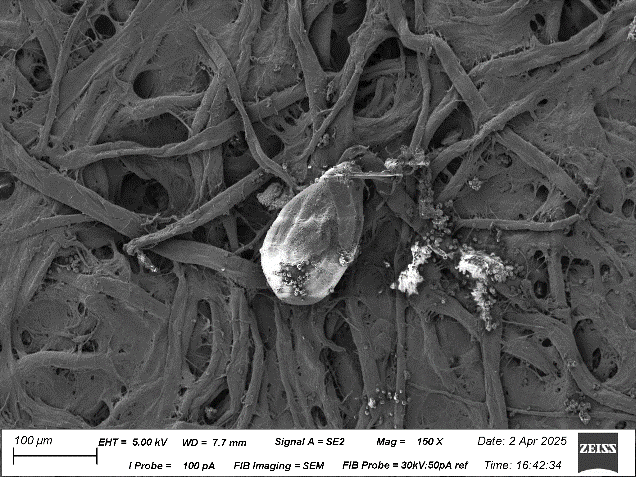 | 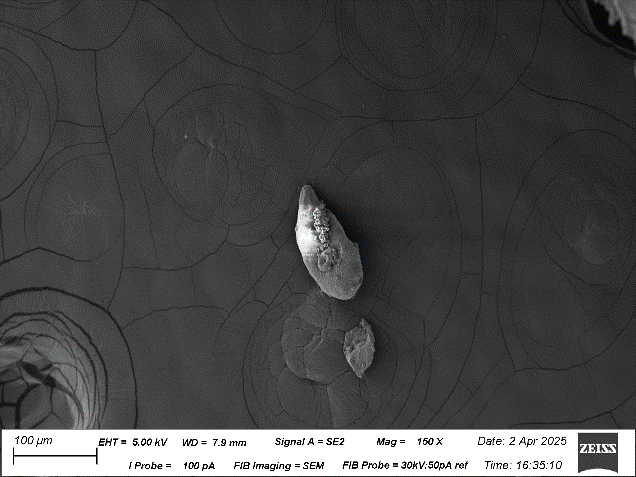 | 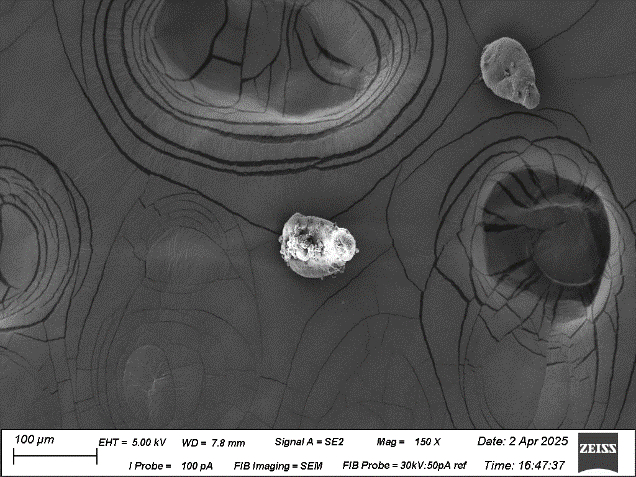 |
| 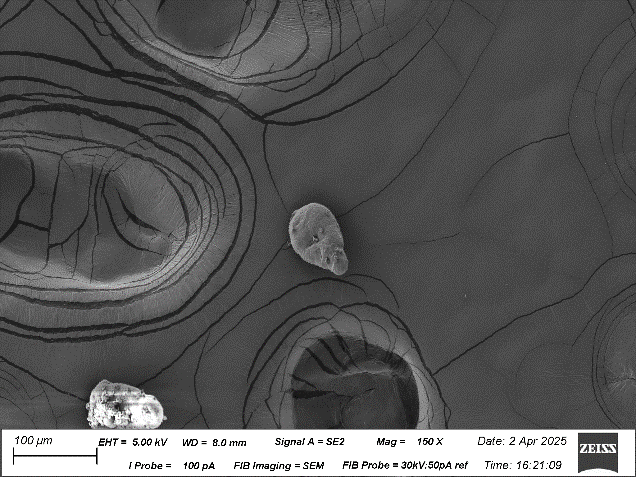 | 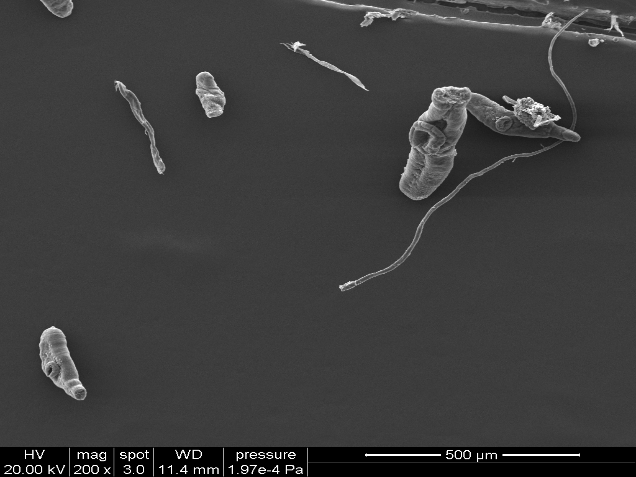 |  |
| Infected + JQ1 Group： |  |  |
| 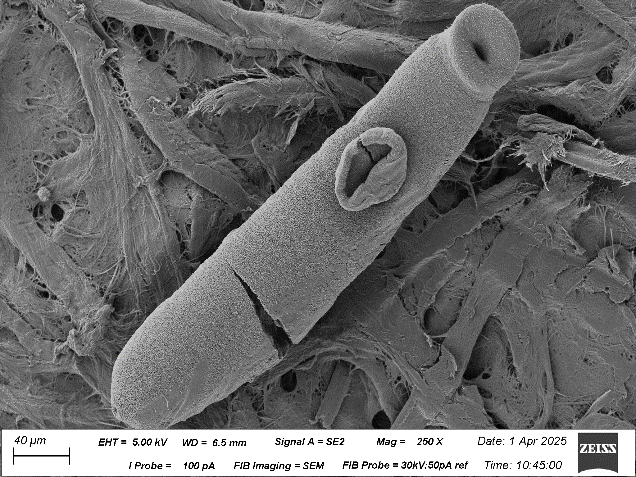 | 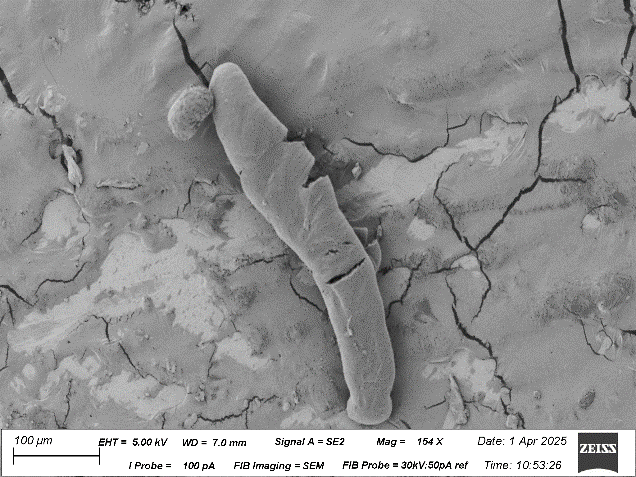 | 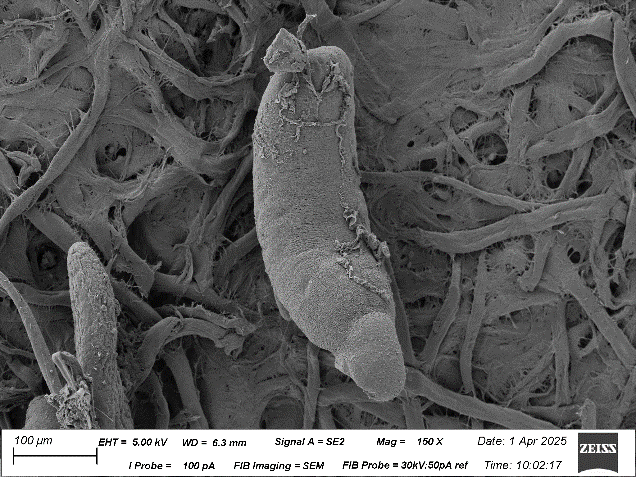 |
| 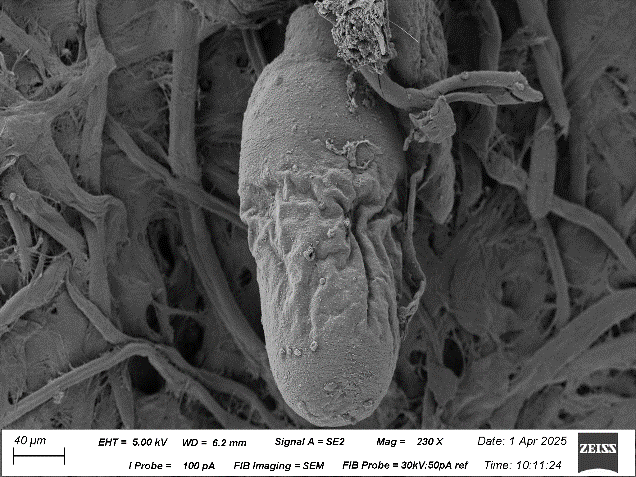 | 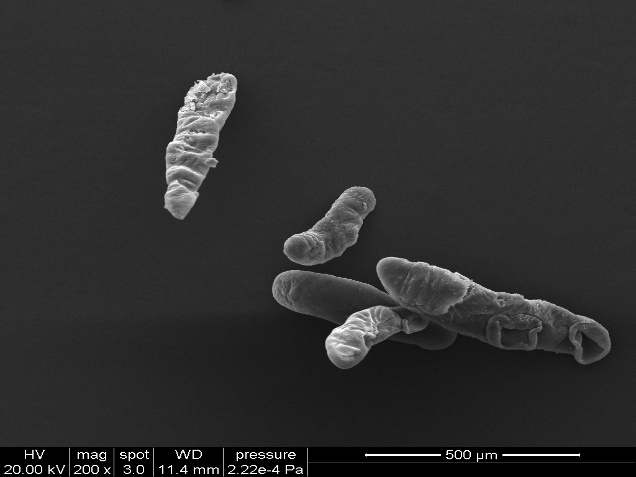 |  |
